# Two Cases of Papillary Thyroid Carcinoma With QTc Prolongation During Selpercatinib Administration: A Case Report

**DOI:** 10.7759/cureus.103878

**Published:** 2026-02-18

**Authors:** Kana Nanjo, Wataru Kida, Ayumi Mori, Yasuhiro Inayoshi, Muneo Nakaya

**Affiliations:** 1 Department of Otolaryngology-Head and Neck Surgery, Tokyo Metropolitan Tama Medical Center, Tokyo, JPN

**Keywords:** papillary thyroid carcinoma (ptc), qtc prolongation, recurrent metastasis, selective ret inhibitor, selpercatinib

## Abstract

Selpercatinib (SEL) is a selective RET (rearranged during transfection) inhibitor that has a good safety profile and potent antitumor effects against RET mutation-positive medullary thyroid carcinoma and RET fusion gene-positive thyroid carcinoma, as demonstrated by the LIBRETTO-001 trial. Its major adverse effects include hypertension, hepatic dysfunction, QTc prolongation, hypersensitivity, and interstitial lung disease. In two patients who received the drug at the study center, SEL demonstrated strong antitumor efficacy but induced QTc prolongation, necessitating a dose reduction or treatment interruption.

The first case involved a 76-year-old female patient with a history of surgery for papillary thyroid carcinoma who had been referred to our department in 2024 due to difficulty with walking caused by right iliac metastases. The patient regained the ability to walk after SEL administration shrank the tumors, but due to QTc prolongation, the treatment was interrupted, and the dosage was reduced.

The second case involved a 54-year-old male patient with papillary thyroid carcinoma and lung metastases who had undergone a total thyroidectomy followed by radioactive iodine therapy in 2019. Subsequently, metastases developed in the intracranial region, bones, liver, muscles, and adrenal glands, leading to the implementation of SEL therapy in September 2024. QTc prolongation occurred on day 14 of treatment. After the treatment was interrupted for one week, the dosage was reduced to 240 mg/day and has been maintained at this level since then. Following this dose adjustment, the liver metastases resolved, and the other metastases decreased in size. No progression was observed at month 14 after the start of the treatment.

In the LIBRETTO-001 trial, QTc prolongation of any grade occurred in approximately 16% of patients, with Grade 3 or higher events reported in about 4%. Reports of QTc prolongation associated with SEL therapy for thyroid cancer remain limited. However, QTc prolongation occurred in both of our patients, suggesting that it may represent a clinically relevant concern in selected individuals. Appropriate dose adjustment and careful monitoring may facilitate continued treatment in selected patients.

## Introduction

Multi-targeted tyrosine kinase inhibitors (MKIs) are normally used to treat unresectable or metastatic differentiated thyroid cancer recalcitrant to radioactive iodine therapy (RAI). RAI-refractory disease refers to tumors that fail to respond to radioactive iodine uptake or that progress despite adequate RAI treatment, a clinical setting in which systemic therapy becomes necessary. In this context, selective, molecularly targeted therapies have expanded the treatment options.

One of the genes implicated in thyroid cancer is RET (rearranged during transfection), which encodes a tyrosine kinase receptor. RET abnormalities, including RET fusions in 10-20% of papillary carcinoma cases and RET mutations identified in more than 95% of hereditary medullary thyroid carcinomas and in approximately 30-50% of sporadic medullary thyroid carcinomas, are the key drivers of thyroid cancer [1,2] and can be detected using next-generation sequencing-based companion diagnostics. Accordingly, RET fusion-positive tumors represent a clinically important molecular subset within papillary thyroid carcinoma, one of the most common types of thyroid cancer.

Selective RET inhibitors, including selpercatinib (SEL) and pralsetinib, have been approved globally for the treatment of RET-related thyroid and lung cancers [3]. In Japan, SEL was approved in 2022 for the treatment of RET fusion-positive thyroid cancer and medullary thyroid carcinoma harboring RET mutations. SEL is an orally administered, highly selective RET inhibitor. Although the frequency of serious adverse events is lower for SEL than for conventional MKIs, the LIBRETTO-001 trial, which established SEL’s antitumor efficacy and safety, identified QTc prolongation, hepatic dysfunction, and events related to hypersensitivity, hypertension, bleeding, and interstitial lung disease as potentially serious adverse events [3].

QTc prolongation reflects delayed ventricular repolarization and is clinically important because it may predispose patients to life-threatening arrhythmias, such as Torsades de Pointes [4]. Against this background, the present report describes two cases of recurrent or metastatic RET fusion-positive papillary thyroid carcinoma (PTC) involving QTc prolongation during SEL therapy. These cases focus on the early occurrence of QTc prolongation, subsequent dose modification, and their impact on clinical outcomes in routine clinical practice. Although SEL has demonstrated durable responses with a favorable safety profile in clinical trials, real-world data on the management of QTc prolongation, particularly in regions where fixed dosing is used, remain limited.

## Case presentation

Adverse events were evaluated using the Common Terminology Criteria for Adverse Events (CTCAE), version 5.0. QTc values were obtained from automated ECG measurements, with the normal range defined as <440 ms for men and <460 ms for women, which are widely used upper limits in clinical cardiology practice [5]. The treatment was administered in accordance with the official, Japanese, prescribing information and guidelines on appropriate use, which prescribe a fixed adult SEL dosage without adjustment for body weight [6]. According to the prescribing information, SEL should be withheld if the QTc interval exceeds 500 ms and may be resumed at a reduced dose once the QTc recovers to <470 ms. Permanent discontinuation is recommended in cases of recurrent QTc prolongation despite dose reduction or when QT prolongation is accompanied by clinical signs suggestive of a serious arrhythmia.

Case 1

**Figure 1:**
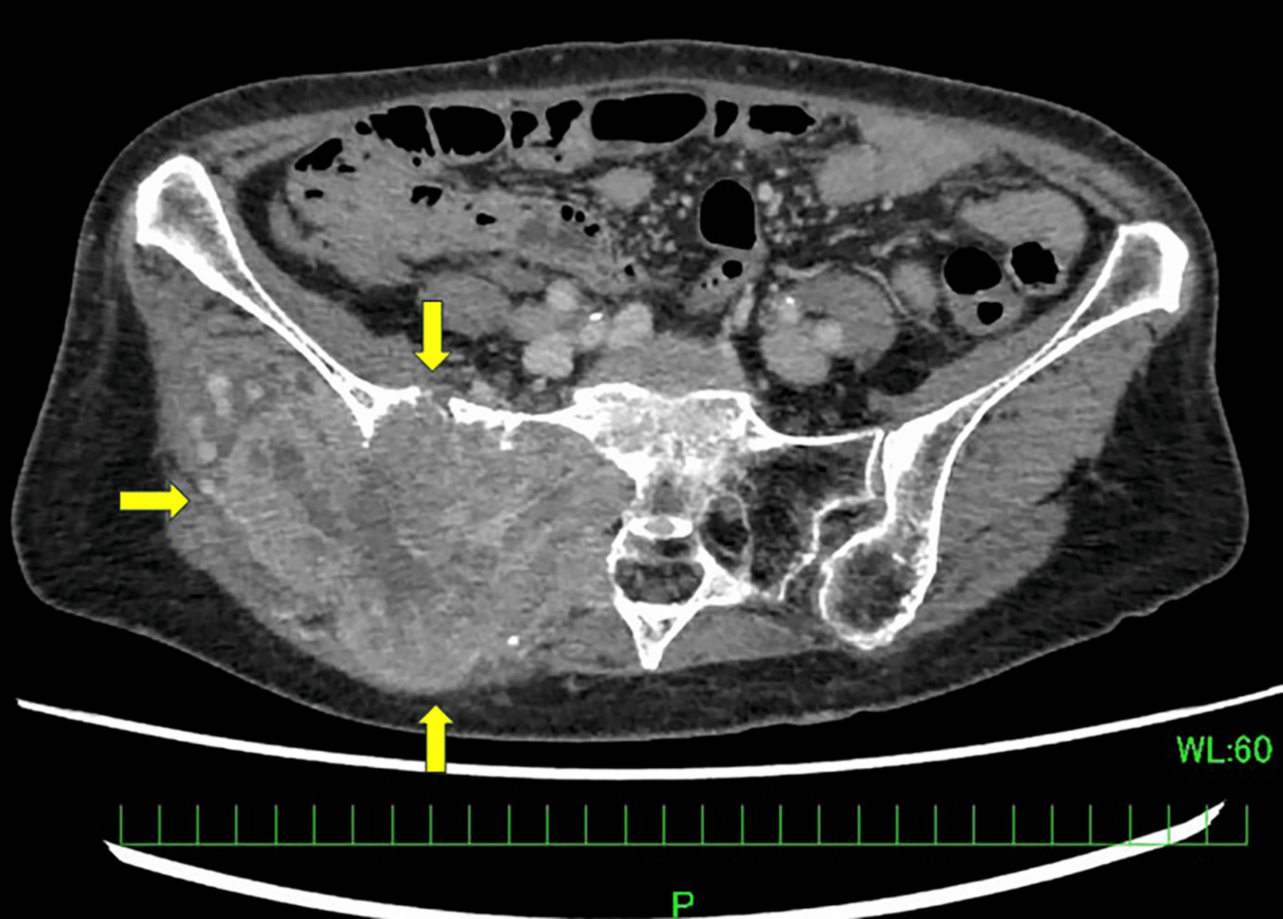
Contrast-enhanced computed tomography (CT) showing a right pelvic bone metastasis (arrows).

A 76-year-old female patient with a history of hypertension and left ventricular hypertrophy underwent a right thyroid lobectomy and right neck dissection for PTC (cT3N0M0 stage II) at a previous facility in 2006. In 2022, multiple pulmonary metastases appeared, for which RAI was recommended; however, she declined the treatment, opting instead for observation. In 2023, swelling developed in her right buttock. Starting in February 2024, she began experiencing numbness in her right lower limb, and metastatic bone tumors developed from the right ilium to the sacrum. A tissue biopsy of the right ilium in June 2024 diagnosed bone metastasis of the papillary thyroid carcinoma. A RET fusion gene was identified using the next-generation sequencing-based multiplex companion diagnostic, the Oncomine Dx Target Test MultiCDx System (ODxTT, Thermo Fisher Scientific, Waltham, MA). Radiation therapy was administered to the metastatic bone tumor in the right ilium in July 2024. SEL administration commenced in August 2024. The baseline serum thyroglobulin (Tg) value was 389 ng/mL. The patient’s body weight at the start of SEL therapy was 42 kg. Baseline contrast-enhanced computed tomography (CT) revealed a right pelvic bone metastasis (Figure 1).

**Figure 2:**
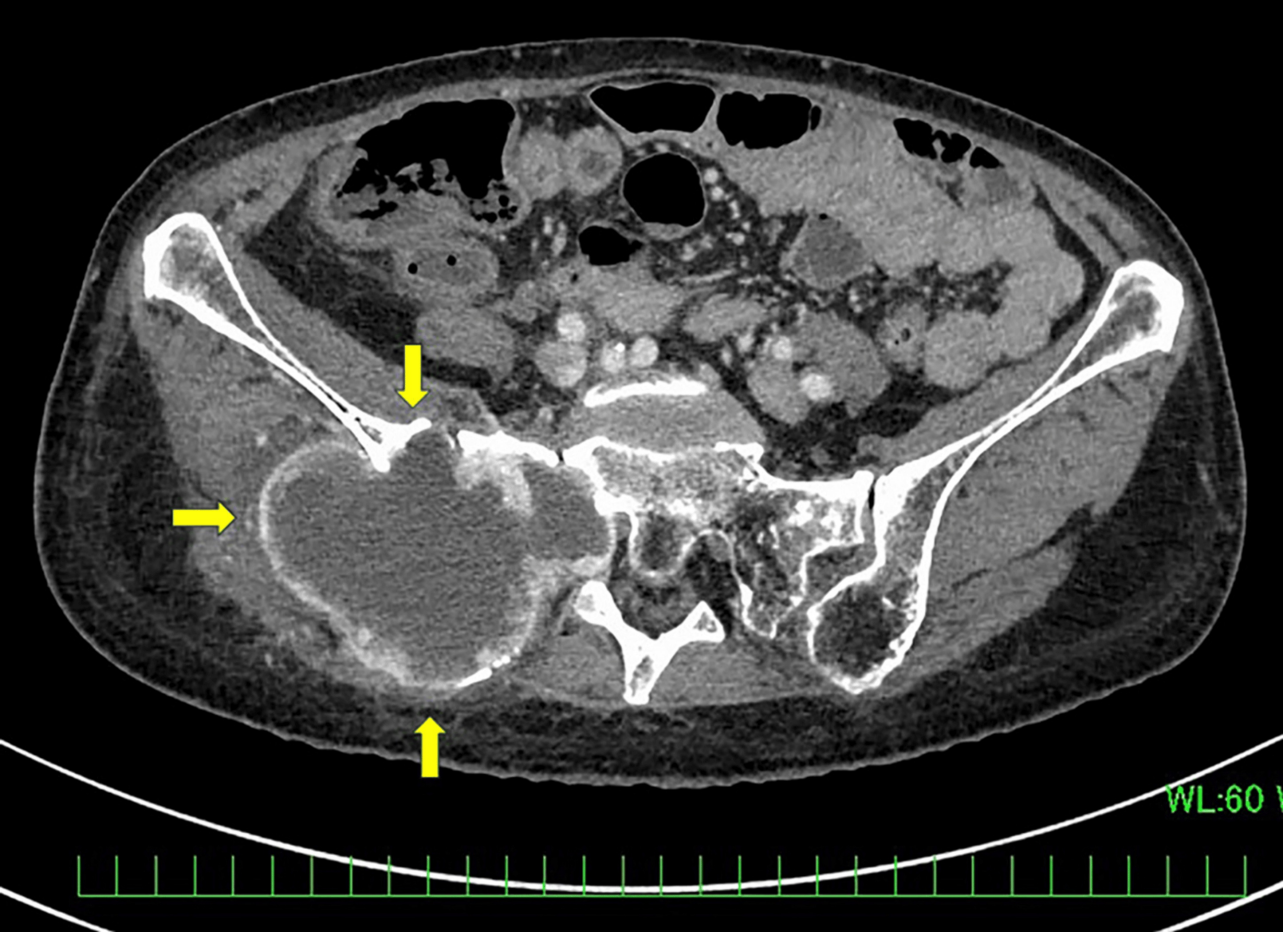
Follow-up imaging of Case 1 after selpercatinib therapy. Contrast-enhanced CT showing a decrease in the size of the right pelvic bone metastasis compared with baseline imaging (arrows).

At admission, the patient had difficulty walking, but the numbness improved sufficiently from Day 8 to permit her to walk. On Day 17, the QTc interval increased from 433 ms at baseline to 504 ms (Grade 3 according to CTCAE, version 5.0). Serum potassium, magnesium, and calcium levels were within normal limits at that time. SEL administration was interrupted in accordance with the prescribing information. The patient had underlying risk factors for QTc prolongation, including advanced age, female sex, hypertension, and left ventricular hypertrophy. The treatment was resumed at 240 mg/day on Day 51 after normalization of the QTc. On Day 73, the QTc interval increased to 523 ms (Grade 3), prompting a second interruption of SEL administration. Contrast-enhanced CT on Day 101, after the second dose interruption, demonstrated tumor shrinkage (Figure 2).

Tg had also decreased significantly to 53.1 ng/mL.

**Figure 3:**
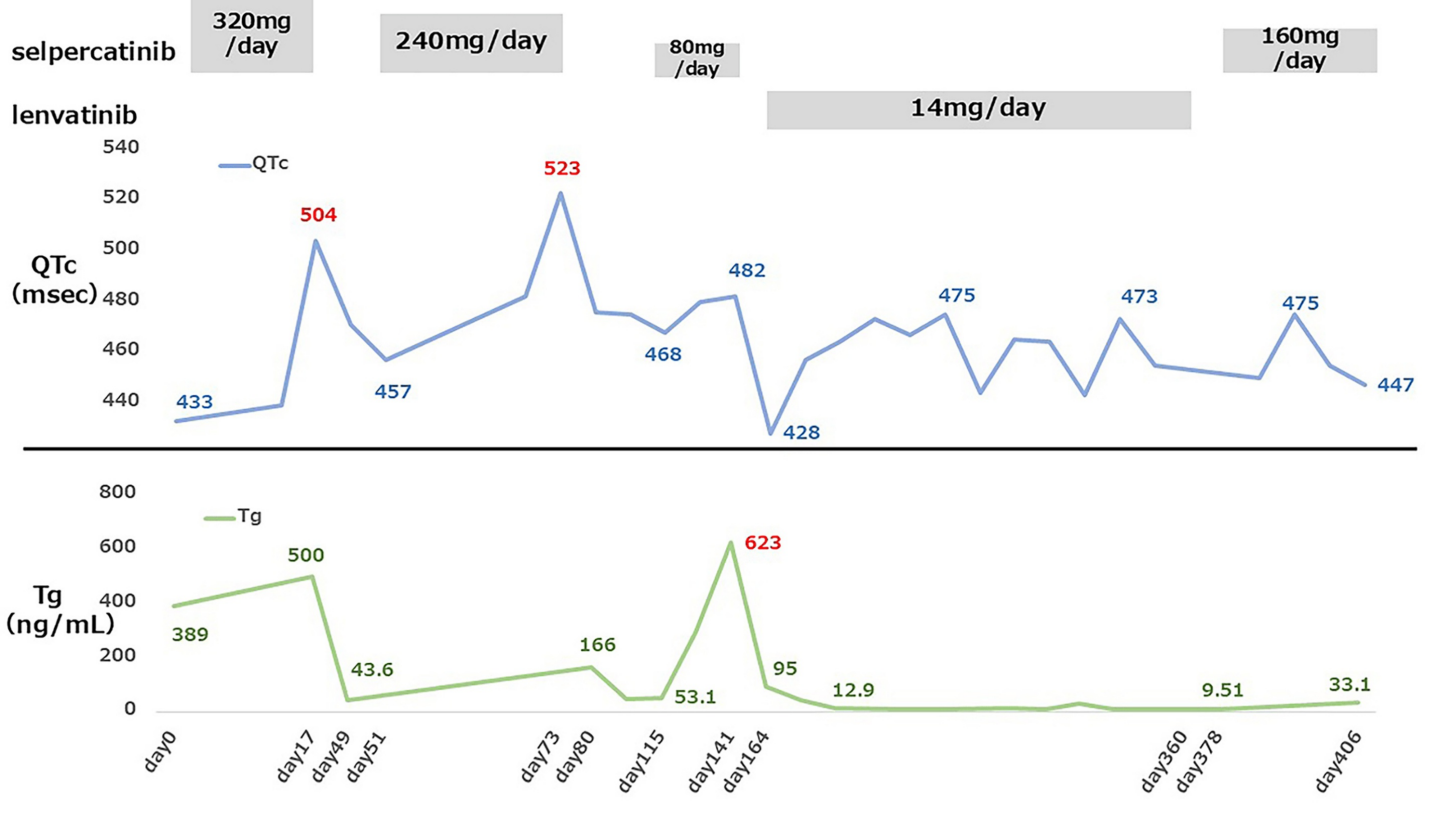
Clinical course of Case 1, illustrating changes in the SEL and lenvatinib dosages, QTc interval, and serum Tg level during treatment. Temporal changes in the SEL and lenvatinib dosages, QTc interval, and serum Tg level are shown. QTc prolongation occurred on Day 17 (504 ms, Grade 3) and Day 73 (523 ms, Grade 3), each requiring treatment interruption. SEL was resumed at reduced doses after QTc normalization. Despite tumor shrinkage and a marked decrease in Tg, recurrent QTc prolongation limited continued therapy, and disease progression ultimately led to treatment discontinuation. SEL, selpercatinib; QTc, corrected QT interval; Tg, thyroglobulin

After QTc normalization, SEL administration was resumed on Day 108 at a reduced dosage of 80 mg/day instead of the two-step reduction to 120 mg/day because of the patient’s history of early QTc prolongation after SEL administration, which had prevented continued treatment. Lenvatinib therapy was initiated in January 2025; however, a subsequent increase in serum Tg level was observed in April 2025, as shown in Figure 3. On Day 141, the Tg level rose. Although the subjective symptoms had not worsened, there was obvious tumor enlargement. Progressive disease (PD) was diagnosed, and SEL therapy was discontinued. Lenvatinib was later administered continuously from January to August 2025, but the metastatic lesions increased, and the right lower limb pain recurred. SEL therapy was therefore resumed at 160 mg/day in September 2025. No QTc prolongation was observed during 30 days of treatment, but disease progression was unable to be controlled. Palliative care at home was initiated in October 2025. The patient experienced no adverse effects other than QTc prolongation during SEL therapy. Figure 3 shows chronological changes in the SEL and lenvatinib dosages, QTc, and serum Tg level.

Case 2

A 54-year-old male patient with an unremarkable medical history was referred to our department in 2019 with the chief complaint of left neck lymph node enlargement. Papillary thyroid carcinoma with multiple lung metastases (cT4aN1M1 stage II) was diagnosed. After undergoing a total thyroidectomy and left neck dissection, he received RAI.

In April 2020, a local recurrence was detected. The recurrence involved the hypopharynx and larynx, for which laryngopharyngectomy was proposed to achieve local disease control. Although laryngopharyngectomy was proposed, the patient declined surgery and opted instead for continued RAI therapy with observation. In 2023, the cancer had metastasized to the thoracic spine and left adrenal gland, and enlargement of the metastases in the lungs and the local recurrence site was also noted. In February 2024, a laryngopharyngectomy and free jejunal reconstruction were performed. By July 2024, the lesion had metastasized to the cervical spine, lumbar spine, sacrum, left ilium, and multiple intracranial sites. Palliative radiotherapy, 30 Gy, was administered to the cervical spine and thoracic metastases.

**Figure 4:**
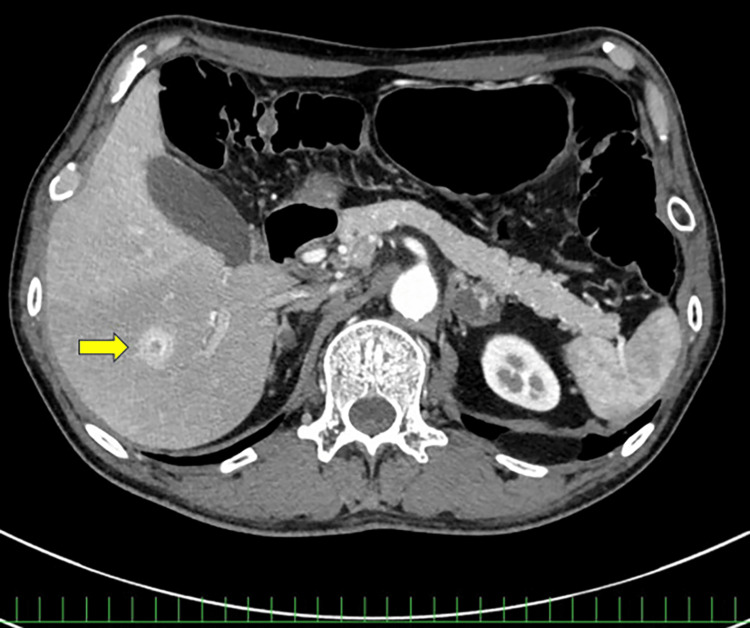
Baseline imaging of Case 2. Contrast-enhanced CT showing liver metastases (arrow).

In September 2024, liver metastases and multiple muscle metastases developed (Figure 4).

The ODxTT found RET fusion gene-positivity, prompting the administration of SEL 320 mg/day in September 2024. The patient’s weight at the start of treatment was 57 kg, and his serum Tg was 122 ng/mL. On Day 13, the QTc interval increased from 419 ms at baseline to 613 ms (Grade 3 according to CTCAE, version 5.0). Serum electrolytes were within normal limits, and no concomitant QT-prolonging medications were identified. SEL therapy was therefore interrupted. After normalization of the QTc, the treatment was resumed on Day 22 at a reduced dosage of 240 mg/day. Case 2 had no established baseline risk factors for QTc prolongation.

**Figure 5:**
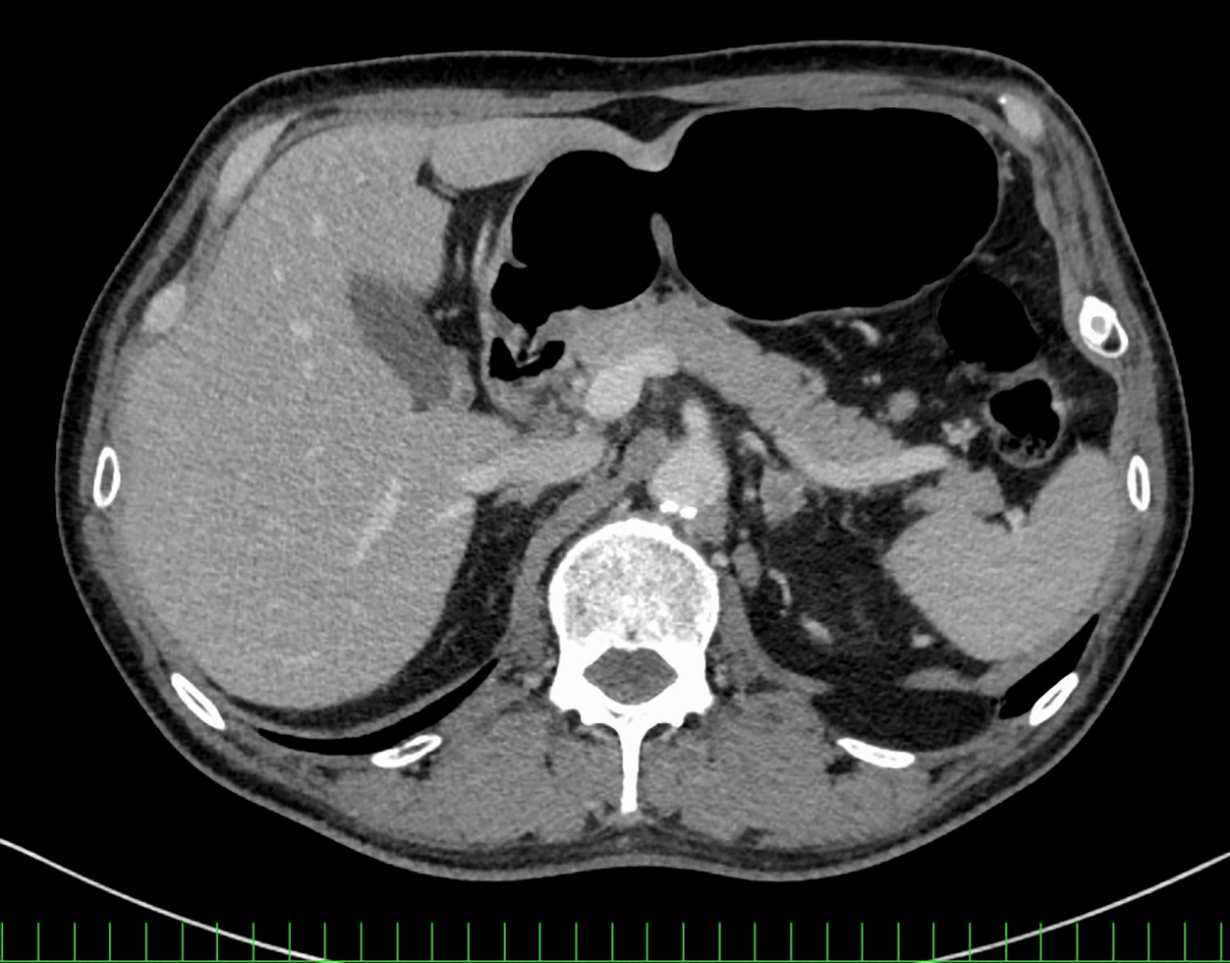
Follow-up imaging of Case 2 after selpercatinib therapy. Contrast-enhanced CT showing no visible liver metastases compared with baseline imaging.

On Day 44, hypertension (systolic blood pressure: 180 mmHg) was noted. An angiotensin-converting enzyme inhibitor (enalapril maleate) was administered. SEL therapy was temporarily discontinued, then resumed on Day 51. Contrast-enhanced CT in December 2024 demonstrated a reduction in the size of the intracranial nodules and multiple pulmonary, muscle, and adrenal metastases, as well as resolution of the liver metastases (Figure 5).

**Figure 6:**
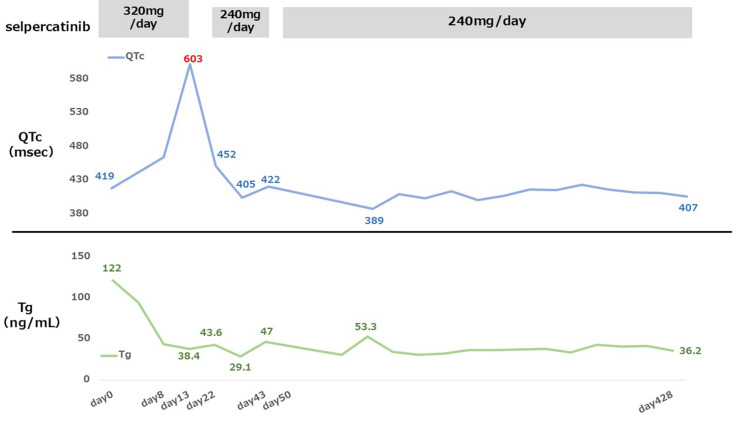
Clinical course of Case 2, illustrating changes in the selpercatinib (SEL) dosage, QTc interval, and serum Tg level during treatment. Temporal changes in SEL dosage, QTc interval, and serum Tg levels are shown. QTc prolongation occurred on Day 13 (613 ms, Grade 3), prompting temporary interruption of SEL therapy. Treatment was resumed at a reduced dose (240 mg/day) after QTc normalization. No recurrent QTc prolongation was observed thereafter, and tumor response was maintained with a sustained decrease in Tg levels. QTc, corrected QT interval; Tg, thyroglobulin

The bone metastases were unchanged. The Tg level decreased gradually after the start of the treatment and has remained below 50 ng/mL to this date. No recurrence of QTc prolongation was noted. SEL 240 mg/day was maintained without interruption, and the disease remains well-controlled. Figure 6 shows chronological changes in the SEL dosage, QTc, and serum Tg level.

## Discussion

Selective RET inhibitors were developed as a molecularly targeted therapy for thyroid cancer and non-small cell lung cancer harboring RET mutations. These mutations are frequently observed in medullary thyroid carcinoma, and nearly all cases of hereditary medullary thyroid carcinoma are known to harbor RET mutations [2]. Moreover, RET fusion genes are present in 10%-20% of papillary thyroid carcinoma (PTC) cases [1], making them some of the most clinically significant driver mutations.

The LIBRETTO-001 trial demonstrated an overall response rate (ORR) of approximately 79% (95% confidence interval (CI) 54-94) in patients with RET fusion gene-positive thyroid cancer and a marked antitumor effect with an ORR of 95.8% (95% CI 78.9-99.9) in a MKI-naive group [7].

Both our patients achieved tumor shrinkage on imaging and improvement in their Tg level, indicating a favorable treatment response. However, they also experienced Grade 3 QTc prolongation, which required treatment interruption and dose reduction to enable the therapy to continue. Subsequently, the patient in Case 2 maintained the therapeutic response after the dose reduction, whereas the patient in Case 1 failed to achieve disease control and required switching to lenvatinib, which produced a different outcome. These contrasting clinical courses highlight heterogeneity in tolerance to SEL-associated QTc prolongation, ranging from durable disease control after dose modification to treatment-limiting toxicity.

QTc prolongation primarily results from the inhibition of the hERG channel in cardiomyocytes [4]. It increases the risk of lethal arrhythmias, such as Torsades de Pointes (TdP), by delaying ventricular repolarization, making it a condition requiring close attention. Major risk factors of drug-induced QTc prolongation include electrolyte abnormalities, such as hypokalemia, hypomagnesemia, and hypocalcemia; a history of heart disease; concomitant use of QTc-prolonging drugs, such as CYP3A4 inhibitors; female sex; age ≥65 years; impaired hepatic or renal function; and a genetic predisposition [8]. However, the mechanism and risk factors of QTc prolongation induced by SEL administration remain unclear.

SEL-induced QTc prolongation is concentration-dependent; the mean increase in QTc coinciding with the maximum plasma concentration (C_max) is 10.6 msec in the context of continued administration of SEL 320 mg/day [9]. The LIBRETTO-001 trial found that QTc prolongation of any grade occurred in approximately 16% of patients, while Grade 3 or higher events were reported in about 4%. For this reason, 2.1% of the cohort required treatment interruption, and 2.5% required a dose reduction, although none required treatment discontinuation [7].

In the present study, SEL was administered in accordance with the official, Japanese prescribing information, which recommends a fixed dosage for adults without adjusting for body weight. However, the dosing recommendations for SEL differ across regions. American guidelines recommend that the dosage for adults and adolescents aged ≥12 years be stratified by body weight, and that patients weighing <50 kg receive a lower dosage [10]. In the present case, one patient weighed 42 kg but received the fixed, adult dosage. The patient’s relatively low body weight may have resulted in higher drug exposure and contributed to the development of QTc prolongation although causality was unable to be established. In addition, other potential risk factors in this patient included advanced age, a history of hypertension, and left ventricular hypertrophy as confirmed by electrocardiography and echocardiography. This discrepancy in the regional dosing recommendations highlights the need to optimize the dosing strategy, particularly for adult patients with low body weight.

Although the Japanese prescribing information provides detailed recommendations for managing QTc prolongation, the management strategies summarized here are based on the American recommendations [10], which have more international currency. Differences in regional dosing strategies may influence drug exposure, particularly in adults with lower body weight. Further pharmacokinetic and real-world studies are warranted to determine whether individualized dosing strategies could improve tolerability without compromising efficacy. Before the start of therapy, electrocardiographic evaluation for QTc and assessment of laboratory parameters, including serum electrolytes, such as potassium, magnesium, and calcium, are recommended. During treatment, periodic electrocardiographic monitoring and assessments of serum electrolytes should be performed in accordance with the patient’s risk of QTc prolongation. If clinically significant QTc prolongation is observed, treatment interruption, dose reduction, or permanent discontinuation of SEL therapy should be considered. If the QTc prolongation improves, the treatment may be resumed at a reduced dosage. However, permanent discontinuation should be considered if the QTc prolongation recurs or persists. In addition, treatment discontinuation is recommended for patients who begin experiencing serious arrhythmia or clinically significant symptoms associated with QTc prolongation, such as dizziness, palpitations, and syncope.

In Case 2, dose reduction and interruption, monthly ECG monitoring, and serum electrolyte assessments were implemented in accordance with the guidelines. The QTc prolongation improved, and the efficacy of the therapy was able to be maintained. However, in Case 1, the frequency of ECG monitoring was increased to every one to two weeks due to the high risk of QTc prolongation. Furthermore, although the guidelines recommend a two-step dose reduction, a three-step dose reduction was implemented; nevertheless, maintaining the therapeutic response proved difficult. These two cases comprise the only instances of SEL therapy in our department, and it is noteworthy that both patients experienced QTc prolongation. While the findings of the present study, based as they are on only two cases at a single institution, require cautious interpretation, QTc prolongation appeared to be a clinically relevant adverse event warranting careful attention in routine practice. Further investigation involving a larger cohort is warranted.

## Conclusions

The present two cases illustrate that SEL can induce meaningful radiologic and biochemical responses in patients with RET fusion-positive PTC. However, QTc prolongation may occur early during treatment and may influence treatment continuation in certain patients. Careful electrocardiographic monitoring and timely dose interruption or reduction may allow therapy to be continued safely in selected individuals. These cases underscore the importance of individualized risk assessment and structured monitoring when administering SEL in routine clinical practice.
